# Anionic Detergents as Eluents for Microscale Isolation of Antigen-Specific Serum Immunoglobulins

**DOI:** 10.3390/bios16010022

**Published:** 2025-12-28

**Authors:** Dmitry Trukhin, Marina Filippova, Alla Tskaeva, Ekaterina Troshina, Dmitry Gryadunov, Elena Savvateeva

**Affiliations:** 1 Center for Precision Genetic Technologies for Medicine, Engelhardt Institute of Molecular Biology, Russian Academy of Sciences, 119991 Moscow, Russiamafilippova@biochip.ru (M.F.); grad@biochip.ru (D.G.); 2 Endocrinology Research Centre, Ministry of Health of Russia, 117292 Moscow, Russia; tskaeva.alla@endocrincentr.ru (A.T.); troshina.ekaterina@endocrincentr.ru (E.T.)

**Keywords:** serum antibodies isolation, anionic detergent, sodium lauroyl glutamate, lauroylsarcosine, sodium dodecyl sulfate, protein array, magnetic beads, multiplex immunoassay

## Abstract

The detailed characterization of antigen-specific serum antibodies is hindered by the lack of efficient, gentle isolation methods. In this context, standard column affinity chromatography, although a powerful purification tool, presents practical challenges, including high antigen consumption and elution conditions that risk inducing antibody polyreactivity, while conventional acidic elution often compromises antibody integrity. This study introduces a novel microscale method for isolating specific immunoglobulins using anionic detergents as mild eluents. We employed antigen-functionalized hydrogel microarrays and magnetic beads as micro-immunosorbents. Among the tested detergents, sodium lauroyl glutamate (SLG) was optimal, achieving up to 78.3% recovery of functional antibodies. The optimized protocol, including recovery via G25-Sephadex gel filtration, effectively isolated specific antibodies from complex serum, retaining 58.5–85.3% of their functional bioactivity. Multiplex immunoassays confirmed the high specificity of the isolated antibodies and the lack of detergent-induced polyreactivity. The method was successfully adapted to isolate both specific antibodies (virus, dietary, and autoimmune) and total IgG, demonstrating versatility across platforms. This work establishes a robust, efficient, and gentle workflow for obtaining high-purity, bioactive antibodies, enabling their subsequent in-depth analysis for research applications.

## 1. Introduction

Disease progression can significantly alter antibody properties through various mechanisms. First, post-translational modifications, such as shifts in glycosylation patterns during diseases, can alter both antigen-binding affinity and effector functions by altering Fc receptor interactions [1]. Current evidence indicates that IgG glycosylation profiles are altered in cancer as well as autoimmune, infectious, and other diseases, affecting both the total pool and certain antigen-specific antibodies [2]. Second, the context of persistent infection itself can drive the formation of polyreactive antibodies, a potential precursor to autoimmunity [3]. Finally, the molecular mimicry hypothesis posits that structural homology between pathogen proteins and self-antigens can misdirect the immune response, leading to the production of autoreactive antibodies [4]. Consequently, investigating antibody glycosylation patterns, polyreactivity, and cross-reactivity is crucial for elucidating the mechanisms that initiate and drive disease progression. However, such analyses are severely constrained by the masking effect of abundant, nonspecific immunoglobulins in serum, which obscure critical changes in low-abundance, antigen-specific fractions. The methodology developed in this work directly addresses this bottleneck by enabling the isolation of purified antigen-specific antibodies, thereby providing a critical tool for elucidating their unique structural and functional characteristics in health and disease.

Affinity chromatography represents the benchmark technique for isolating specific proteins from complex biological mixtures. This method is particularly valuable for purifying serum antibodies, as it yields isolates that faithfully retain the native immunoglobulin classes, glycosylation profiles, and antigen-binding affinity. Despite its conceptual advantages, conventional column affinity chromatography presents significant practical limitations for the isolation of low-abundance specific antibodies. The method requires large quantities of purified, stable antigens for sorbent synthesis, creating a major cost barrier. The subsequent elution step further complicates the process, as it leads to sample dilution and frequent protein loss during concentration and buffer exchange. Consequently, the column chromatographic isolation of specific antibodies from multiple samples remains a labor-intensive, time-consuming, and resource-heavy endeavor. Alternative approaches have circumvented the use of columns by utilizing other platforms, such as the Western blot membrane strips employed by Kurien et al. [5] or the immunoassay plates used by Mendis et al. [6]. Meanwhile, advances in magnetic-particle-based technologies have facilitated the development of miniaturized methods for protein isolation from complex mixtures, significantly expanding their application potential [7].

Acidic buffers (pH 2.5–3.0) remain a common choice for eluting antibodies from antigen-affinity matrices due to their simplicity and low cost. However, even with brief exposure and immediate neutralization, the low pH can cause irreversible protein degradation, rendering the antibodies unsuitable for functional activity studies. Beyond degradation and aggregation [8], a more insidious consequence is the induction of polyreactivity, whereby previously monospecific antibodies begin to bind nonspecifically to multiple unrelated antigens following acid exposure [9,10,11].

Mild elution agents offer a gentler alternative for dissociating antibodies from affinity matrices. While the common anionic detergent sodium dodecyl sulfate (SDS) is a potent denaturant that irreversibly disrupts protein structures, several of its analogs interact with proteins in a milder, reversible manner [12]. Detergents like sodium lauroyl sarcosinate (Sarkosyl) and sodium lauroyl glutamate (SLG) can solubilize proteins from inclusion bodies with minimal denaturation [13,14]. It is hypothesized that these milder detergents engage in limited protein penetration, primarily interacting with hydrophobic surface regions to induce transient conformational changes without causing complete denaturation. When applied to antigen–antibody complexes, this mechanism enables complex dissociation while minimizing damage to the antibody’s native structure. Critically, when detergent residues are removed following antibody isolation, bioactivity is substantially recovered, representing a key advantage over conventional denaturing elution methods.

This study aimed to develop a microscale workflow for isolating antigen-specific serum antibodies using array- and bead-based platforms, with anionic detergents employed as a gentle elution strategy to preserve antibody functionality.

## 2. Materials and Methods

### 2.1. Serum Samples

Serum samples were collected from a cohort of patients with autoimmune diseases and control subjects [15]. From this collection, ten samples that were multipositive for IgG against bovine serum albumin (BSA), cytomegalovirus (CMV) protein pp150, and/or thyroglobulin (Tg) were selected based on a multiplex assay.

This study was conducted in accordance with the Declaration of Helsinki and approved by the local ethics committee of the Endocrinology Research Centre, Ministry of Health of Russia, Moscow, Russia (protocol No. 14 and date of approval 29 July 2022, Supplementary File S1). After serum samples were collected from patients, they were aliquoted and stored at −80 °C (long-term).

### 2.2. Fabrication of Micro-Immunosorbents

#### 2.2.1. Capture Arrays

Hydrogel capture arrays with immobilized proteins were manufactured via co-polymerization immobilization following a previously described protocol [16]. The following proteins were used for immobilization: BSA (A7030, Sigma, St. Louis, MO, USA), recombinant thyroglobulin (8RTG4, HyTest, Turku, Finland), recombinant cytomegalovirus pp150 protein (RE003, Xema Co., Ltd., Moscow, Russia), and recombinant human insulin protein (ab123768, Abcam, Cambridge, UK). For method development, a capture array with immobilized insulin was employed in combination with Cy5.5 fluorescently labeled monoclonal antibodies against insulin (RC3A6, HyTest, Finland). The array layout was identical for all capture arrays, regardless of the antigen used (Figure S1).

#### 2.2.2. Magnetic Beads

Except for insulin, the same antigens used to fabricate the microarray were used to isolate specific antibodies. The following proteins were immobilized: bovine serum albumin (BSA; A7030, Sigma, St. Louis, MO, USA), recombinant human thyroglobulin (8RTG4, HyTest, Turku, Finland), and recombinant cytomegalovirus pp150 protein (RE003, Xema Co., Ltd., Moscow, Russia). Proteins were covalently immobilized on amino-functionalized magnetic nanoparticles (iron oxide, 300–400 nm; K0501, Sileks, Moscow, Russia) using glutaraldehyde crosslinking, following the manufacturer’s protocol. In brief, 100 μL of the magnetic particle solution (5 mg/mL) was washed and resuspended in 250 μL of PBS containing 0.01% Tween20 (PBSt), and 250 μL of 25% glutaraldehyde was added; the mixture was then incubated in the dark for 3 h with gentle rocking. After four washes with PBSt, the particles were resuspended in 500 µL of PBSt, mixed with 100 µL of protein (0.2 mg/mL) in PBSt, and incubated overnight with gentle rocking, after which they were washed four times with PBSt again and resuspended in 100 µL of PBSt.

Total IgG isolation was performed using ready-to-use, commercially available protein G-coated magnetic beads (786-904, Geno Technology, St. Louis, MO, USA).

### 2.3. Anionic Detergents

The following detergents were used: sodium lauroyl glutamate (sc-495823, Santa Cruz Biotechnology, Inc., Dallas, TX, USA), N-Lauroylsarcosine (Sodium salt) (L-5125, Sigma, St. Louis, MO, USA), and sodium dodecyl sulfate (SB-GC204005-01, ServieceBio, Wuhan, China). Stock solutions were prepared differently depending on the anionic detergent. SLG was first dissolved in milli-Q water (up to 20%), followed by gradual titration with a 10 M NaOH solution until clear. SDS was dissolved in water with gentle stirring and heating (~50 °C). Sarkosyl was readily soluble in water. The stock solutions were stored at room temperature and warmed before use if necessary. Working solutions of the detergents (SDS, 0.006–0.5%; Sarcosyl, 0.25–10.0%; SLG, 0.25–10.0%) were prepared fresh in milli-Q water immediately prior to use.

### 2.4. Research Methodology

**Figure 1:**
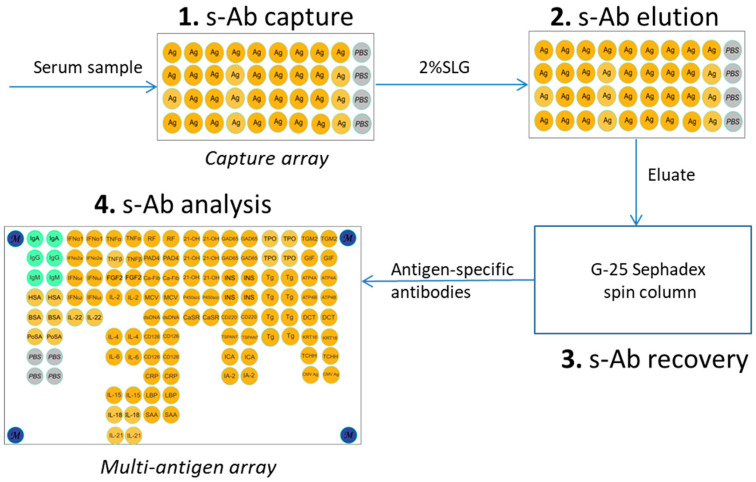
The workflow for the microscale isolation of antigen-specific antibodies from human serum. The diagram illustrates the three-stage process using micro-immunosorbents (microarrays or magnetic beads) and anionic detergent elution. The stages are (1) capture of specific antibodies (s-Ab) from serum by the immobilized antigen; (2) gentle elution using sodium lauroyl glutamate (SLG) to dissociate the complexes; (3) purification via gel filtration to remove the detergent and recover functional antibodies; (4) analysis of isolated antibodies.

The procedure involved three stages: (1) incubation of a serum sample with the micro-immunosorbent (capture array or magnetic beads) to facilitate the formation of specific complexes between the immobilized antigens and target antibodies; (2) elution of the captured antibodies using a non-denaturing anionic detergent; and (3) purification of the isolated antibodies from the eluting agent to restore their antigen-binding activity for further analysis (Figure 1).

#### 2.4.1. Antigen-Specific Immunoglobulin Isolation with Protein Arrays

The serum sample was diluted 1:50 in immunoassay buffer, and 100 µL of the dilution was applied to the array. Following an overnight incubation at 37 °C, the array was washed with PBSt for 20 min, rinsed with water, and dried. Subsequently, 100 µL of a freshly prepared eluent containing an anionic detergent was added, followed by incubation for 1 h at 37 °C. The final eluate was then carefully collected with a pipette.

#### 2.4.2. Antigen-Specific Immunoglobulin Isolation with Magnetic Beads

Antibodies were isolated from 20 µL of blood serum using 50 µL of a magnetic particle suspension with immobilized protein. Magnetic particles were blocked for 1 h in 100 μL of EveryBlot Blocking Buffer (Bio-Rad Laboratories, Hercules, CA, USA), washed, and resuspended in 500 μL of PBSt, to which a blood serum sample (20 μL) was added, incubated for 1 h with gentle rocking, washed three times with 500 μL PBSt, and resuspended in 100 μL of PBSt. Next, 100 μL of Elution Buffer (0.1 M Gly-HCL, pH 2.5 or 2% SLG in PBS) was added to the particles and incubated for 10 min, with periodic mixing by pipetting. If acidic elution was performed, the eluate was immediately neutralized with 20 μL of 1 M Tris-HCl (pH 8.5).

#### 2.4.3. Total IgG Isolation with Magnetic Beads

Patient serum samples (20 µL) were added to tubes containing 50 µL of magnetic beads coated with Protein G (786-904, Geno Technology, St. Louis, MO, USA) and incubated for 1 h at room temperature with end-over-end rotation. Following incubation, the beads were washed three times with 200 µL of binding buffer, with the supernatant removed after each wash. After the final wash, 100 µL of elution buffer was added to the magnetic beads and incubated for 10 min, with periodic resuspension. When an acidic elution buffer (0.1 M Gly-HCL, pH 2.5) was used, the eluate was neutralized with 1 M Tris-HCl buffer (pH 8.5) at a ratio of 1:5.

### 2.5. Recovery of Individual Antigen-Specific Immunoglobulins

#### 2.5.1. Gel Filtration

The obtained eluates (100 µL) were applied to spin columns (7326204, Bio-Rad Laboratories, USA) filled with Sephadex G-25 coarse (17-0034-02, GE Healthcare, Chicago, IL, USA) and pre-equilibrated with PBS and centrifuged at 1500× *g*. The resulting probe was used for further analysis.

#### 2.5.2. Dialysis

The obtained eluates (100 µL) were dialyzed using Slide-A-Lyzer MINI dialysis devices (3.5 kDa MWCO; Thermo Fisher Scientific, Carlsbad, CA, USA) against three PBS changes (pH 7.2, 300 mL) under constant stirring at RT.

### 2.6. Analysis of Isolated Immunoglobulins

#### 2.6.1. SDS-PAGE and Western Blot

The obtained samples were separated by electrophoresis under denaturing conditions using a 10% resolving gel. Protein bands were visualized by staining with either Coomassie R-250 (786-495, Geno Technology, USA) or silver nitrate (G2080, Servicebio, Wuhan, Hubei, China). The proteins were then transferred to a PVDF membrane (1620262, Bio-Rad Laboratories, USA). Following the transfer, the membrane was blocked overnight with EveryBlot Blocking Buffer (12010020, Bio-Rad Laboratories, USA). The membrane was incubated for one hour with polyclonal goat anti-human IgG antibodies (31163 Invitrogen, Thermo Fisher Scientific, Carlsbad, CA, USA) and then for one hour with horseradish peroxidase-conjugated rabbit anti-goat IgG antibodies (S0010, Affinity Biosciences, Loveland, CO, USA). After washing with PBS containing 0.01% Tween 20, the immune complexes were visualized using the Clarity™ Western ECL substrate kit (1705061, Bio-Rad Laboratories, USA).

#### 2.6.2. Multiplex Assay on Multi-Antigen Array

**Figure 2:**
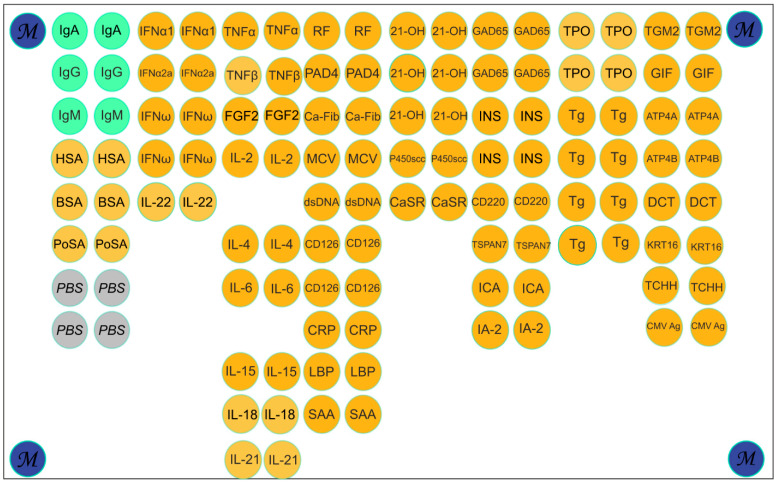
Multi-antigen array layout. The microarray consisted of 120 elements, including 6 control elements with human immunoglobulins A, G, and M (IgA, IgG, IgM); 4 empty hydrogel elements without immobilized proteins (PBS); and 4 elements with a fluorescent marker (M). The catalog number and the source for each of the immobilized proteins are listed in Table S1. Abbreviations: IgA—immunoglobulin A; IgG—immunoglobulin G; IgM—immunoglobulin M; HSA—human serum albumin; BSA—bovine serum albumin; PoSA—albumin from porcine serum; PBS—empty gel; IFNa1—Interferon alpha; INFa2a—Interferon alpha 2a; IFNω—Interferon omega; IL-22—Interleukin 22; TNFα—tumor necrosis factor alpha; TNF-β—tumor necrosis factor beta; FGF2—fibroblast growth factor 2; IL-2—Interleukin 2; IL-4—Interleukin 4; IL-6—Interleukin 6; IL-15—Interleukin 15; IL-18—Interleukin 18; IL-21—Interleukin 21; RF—Fc fragment from papain-digested human IgG (heavy-chain dimer); PAD4—Peptidylarginine Deiminase 4; Ca-Fib—Carbamylated Human Fibrinogen; MCV—Citrullinated Vimentin; dsDNA—double-stranded DNA; CD126—sIL-6 Receptor α; CRP—C-reactive protein; LBP—lipopolysaccharide binding protein; SAA—serum amyloid A1; 21-OH—cytochrome P450c21; P450scc—cholesterol side-chain cleavage enzyme; CaSR—Ca-sensing receptor; GAD-65—glutamic acid decarboxylase 65 kDa; INS—insulin human; CD220—insulin receptor; TSPAN7—Tetraspanin-7; ICA—islet cell autoantigen 1; IA-2—tyrosine phosphatase-like autoantigen; TPO—thyroid peroxidase; Tg—Thyroglobulin; TGM2—tissue transglutaminase 2; GIF—Gastric Intrinsic Factor; ATP4A—alpha subunit of the parietal cell H+/K+-ATPase; ATP4B—beta subunit of the parietal cell H+/K+-ATPase; DCT—dopachrome delta-isomerase; KRT16—Keratin 16; TCHH—Trichohyalin; CMV Ag—cytomegalovirus pp150 protein.

Hydrogel multi-antigen arrays with immobilized proteins were manufactured via co-polymerization immobilization following a previously described protocol [16]. The multi-antigen array contained 120 elements with 58 immobilized proteins, corresponding to 47 unique proteins. The proteins for the multi-antigen array are listed in Table S1. The microarray layout is shown in Figure 2.

For the multiplex immunoassay, both original blood serum samples and specific antibodies isolated from them were used. Serum samples were diluted at a 1:50 ratio, and isolated antibodies were analyzed without dilution. IgG antibodies targeting 60 immobilized proteins were detected using a previously developed assay [16]. Microarrays were blocked with 1% polyvinyl alcohol (PVA) in phosphate-buffered saline (PBS, pH 7.4) at room temperature (RT) for 1 h. Samples (100 µL) were applied to the microarrays. Following an overnight incubation at 37 °C, the arrays were washed with PBS containing 0.1% Tween-20 for 20 min. Antigen–antibody complexes were then detected using a fluorescently labeled anti-human IgG antibody (31163, Invitrogen, Thermo Fisher Scientific, Carlsbad, CA, USA) during two hours of incubation at 37 °C. Finally, the microarrays were washed with PBS containing 0.1% Tween-20 for 30 min, rinsed with buffer, and dried prior to scanning.

Fluorescence images of microarrays were obtained using a proprietary laser-excited microarray analyzer [17]. Signals were quantified with proprietary software. For each group of n elements containing identical antigens, the resulting signal (I_n_) was calculated as the mean fluorescence intensity of the corresponding spots. To account for variations in total IgG concentration, normalization was performed by incorporating the individual background signal from empty gel elements (I_n_/I_ref_) into the analysis for serum samples. Elution efficiency (%) was calculated as the ratio of the median signal from the corresponding array elements after elution to their initial signal. Recovery efficiency (%) was calculated as the ratio of the median signal from the corresponding array elements for isolated specific antibodies to the signal obtained from the same element group during the initial sample analysis.

### 2.7. Direct Analysis of Antibodies on the Microarray

Cy5.5-labeled antibodies were diluted in PBS containing 0.14% PVA and 0.14% PVP. A 100 µL volume of the antibody solution (at concentrations ranging from 500 ng/mL to 2.0 µg/mL) was applied to the microarrays. After a two-hour incubation at 37 °C, the microarrays were washed with PBS containing 0.1% Tween 20 for 30 min, rinsed with distilled water, and dried. The following antibodies were used: monoclonal anti-insulin (clone RC3A6, HyTest, Finland), polyclonal anti-BSA (A11133, Invitrogen, Thermo Fisher Scientific), and goat anti-human IgG (31163, Invitrogen, Thermo Fisher Scientific).

## 3. Results

### 3.1. Selection of Anionic Detergents as Eluents

This study describes a mild elution method for recovering antibodies from an immunosorbent while preserving their specific antigen-binding activity. The approach utilized micro-immunosorbents, such as microarrays or magnetic particles, containing microquantities of covalently immobilized antigens. Specific antibodies were isolated using a three-stage protocol. First, the sample was incubated with the micro-immunosorbent for specific antibody capture. Next, the bound antibodies were eluted with a non-denaturing anionic detergent. Finally, the eluate was purified to remove the detergent and restore the antibody’s functional activity for subsequent analysis (Figure 1). To optimize the elution protocol, a series of experiments was conducted using protein microarrays to evaluate key parameters, including the anionic detergent and its concentration, buffer composition, elution time, temperature, and the subsequent detergent removal method. The experimental setup employed a capture microarray with immobilized insulin and fluorescently labeled (Cy5.5) monoclonal anti-insulin antibodies. The assay was designed in a direct format, forming a specific “immobilized insulin–anti-insulin antibody” complex within microarray elements (100 ± 20 µm in diameter). This design allowed for the direct fluorescence monitoring of three key processes: the initial capture of antibodies, their desorption during elution, and the functional re-binding of the isolated antibodies to a fresh microarray.

**Figure 3:**
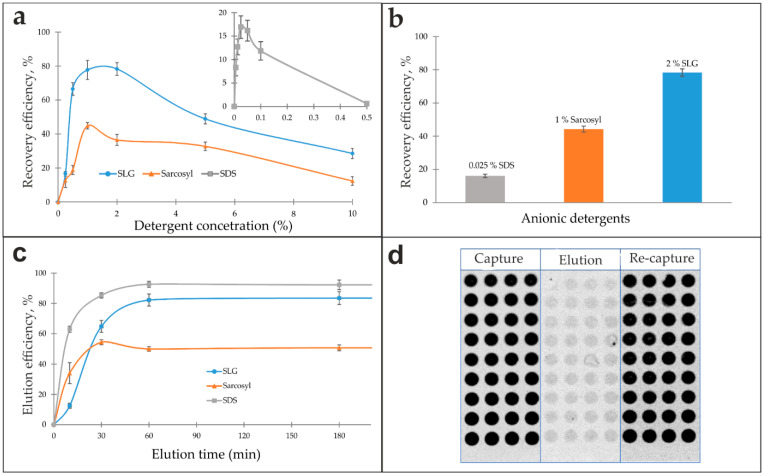
Evaluation of anionic detergents as eluents for antibody isolation. (**a**) Antibody recovery efficiency as a function of anionic detergent concentration. Data for SDS are presented in the inset. (**b**) Comparison of the maximum elution efficiency achieved by optimal concentrations of SDS, Sarcosyl, and SLG. (**c**) Effect of eluent exposure time on elution efficiency. (**d**) Assessment of the matrix effect during antibody isolation from serum using a capture microarray. The schematic illustrates the experimental workflow: initial antibody capture from serum (“Capture”), elution with 2% SLG (“Elution”), and re-binding of the purified antibodies (“Re-capture”). Fluorescence images correspond to each step.

Solutions of anionic detergents—SDS (0.006–0.5%), Sarcosyl (0.25–10.0%), and SLG (0.25–10.0%)—were evaluated as eluents. The antibody recovery efficiency as a function of detergent concentration exhibited a distinct bell-shaped profile for all tested agents, as shown in Figure 3a. Each detergent demonstrated a well-defined optimal concentration that yielded maximum recovery, with lower or higher concentrations diminishing efficiency. Subsequent analysis of the eluted antibodies’ functional integrity in a re-capture assay revealed maximum bioactivity recoveries of 16.9% for 0.025% SDS, 44.8% for 1% Sarcosyl, and 78.3% for 2% SLG, relative to the initial input (Figure 3b).

The elution kinetics for 0.025% SDS, 1% Sarcosyl, and 2% SLG solutions were evaluated over a time range of 10 min to 16 h. After a 180 min elution time, the response curves plateaued (Figure 3c). An elution time of one hour was determined to be optimal, representing a saturation point beyond which no further increase in efficiency was observed. Shorter incubation times were insufficient for complete dissociation of the antigen–antibody complexes. The process was found to be largely temperature-independent, as similar recovery yields were obtained at 4 °C, room temperature, and 37 °C. A temperature of 37 °C was selected for the standard elution protocol to maintain methodological consistency with the serum incubation and antibody assay steps, which were performed at this temperature.

The resulting eluates were purified by either dialysis or gel filtration using Sephadex G-25 spin columns. Gel filtration was selected as the primary method due to its comparable efficiency to dialysis, coupled with its significantly faster processing and highly reproducible eluate volumes. With the optimized protocol (2% SLG, 37 °C for 1 h, followed by gel filtration), the model system using fluorescently labeled anti-insulin antibodies spiked into serum (500 ng/mL) achieved an 80% desorption efficiency while preserving 78% of the antibodies’ specific bioactivity, as confirmed by a re-capture assay on a new microarray (Figure 3d). Eluates that did not undergo the purification and recovery procedure exhibited a complete loss of specific binding activity.

### 3.2. Microscale Isolation of Serum Antibodies Using Anionic Detergent Elution: Proof of Concept

Ten serum samples containing multiple autoantibodies, specifically IgG antibodies targeting thyroglobulin (Tg), bovine serum albumin (BSA), and cytomegalovirus (CMV) protein pp150, were selected from a previously characterized cohort [15] based on a multiplex assay. Following the developed protocol (Figure 1), the samples were incubated on capture microarrays (Figure S1) with the corresponding immobilized antigens. Subsequently, captured antibodies were eluted using a 2% SLG solution. The resulting eluate was purified via gel filtration on a spin column and then analyzed on a multi-antigen microarray (Figure 2). For each sample (*n* = 10) and each target antigen (*n* = 3), the isolation procedure yielded specific signals on the multi-antigen array, with no detectable cross-reactivity. The procedure successfully preserved 58.5–85.3% of the antibodies’ specific bioactivity, depending on the initial antibody titer and the specific capture antigen used. The results for these samples are presented below to demonstrate the practical application of the method.

**Figure 4:**
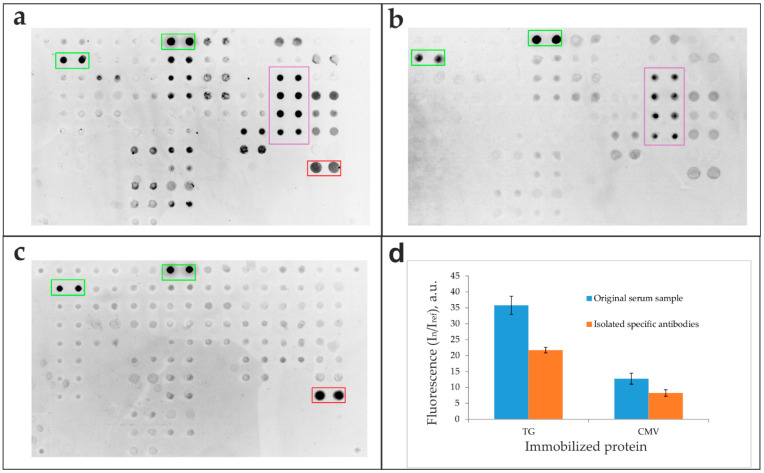
Multi-antigen microarray analysis of original serum and affinity-isolated antibodies. (a-c) Fluorescence images of the microarray after application of (**a**) the original serum sample and antibodies specifically eluted from capture microarrays functionalized with (**b**) thyroglobulin (Tg) and (**c**) cytomegalovirus (CMV) antigens. (**d**) Fluorescence signals (In/Iref) from the microarray elements after analysis of the original serum sample and isolated specific anti-Tg and anti-CMV antibodies. Legend: Green rectangle—binding of the goat anti-human IgG detection antibody to control elements containing human IgG; red rectangle—antibody binding to elements containing CMV; purple rectangle—antibody binding to elements containing Tg.

To validate the isolation specificity, the original sample (Figure 4a) and antibodies eluted from Tg- and CMV-specific capture microarrays (Figure 4b,c, respectively) were analyzed in parallel on a multi-antigen microarray. The corresponding fluorescence signals from the microarray elements are presented in Figure 4d.

### 3.3. Evaluation of the Developed Elution Method Using Magnetic Particles as an Immunosorbent

The developed elution protocol was further validated using magnetic beads as an alternative immunosorbent platform. Three proteins (Tg, BSA, and CMV pp150) were covalently immobilized onto amino-functionalized magnetic particles via glutaraldehyde crosslinking. To ensure direct comparability with the microarray experiments, identical antigen preparations were used for both the magnetic beads and the microarray surfaces. Antigen-specific antibodies from serum samples were isolated using two distinct elution methods: (1) a standard manufacturer’s protocol involving acidic elution (Glycine-HCl, pH 2.5) followed by immediate neutralization; (2) the newly developed mild elution protocol using an anionic detergent (2% SLG) followed by purification on Sephadex G-25 spin columns.

**Figure 5:**
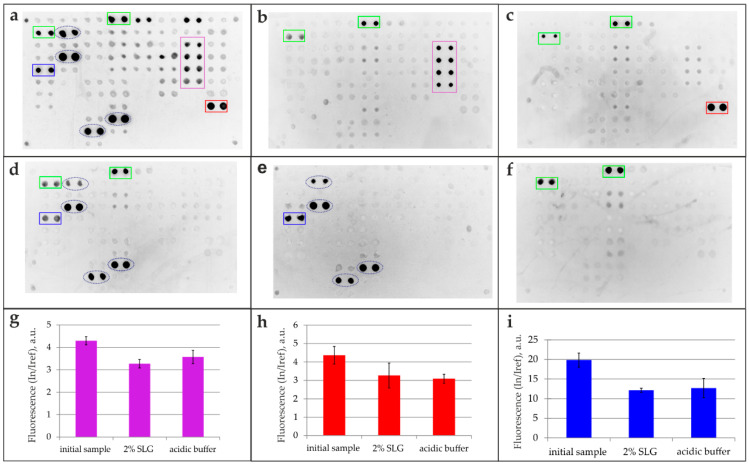
Isolation of antigen-specific antibodies using magnetic beads. Antibody specificity was analyzed using a multi-antigen microarray. (**a**) Fluorescence image of the original serum sample. (**b**–**d**) Fluorescence images after an assay of antibodies isolated via 2% SLG elution from magnetic beads functionalized with (**b**) thyroglobulin (Tg), (**c**) cytomegalovirus (CMV), and (**d**) bovine serum albumin (BSA) antigens. Control experiments include (**e**) application of fluorescently labeled anti-BSA antibodies to assess assay interference and direct application of fluorescently labeled goat anti-human IgG detection antibody (**f**). (**g**–**i**) Comparison of fluorescent signals from microarray elements for the initial sample and antibodies isolated via 2% SLG or acidic (Glycine-HCl, pH 2.5) elution. Signals for immobilized Tg (**g**), CMV pp150 (**h**), and BSA (**i**) antigens are shown. Legend: Green rectangle—binding of the goat anti-human IgG detection antibody to control elements containing human IgG; red rectangle—antibody binding to elements containing CMV; purple rectangle—antibody binding to elements containing Tg; blue rectangle—antibody binding to elements containing BSA; blue dashed oval—antibody binding to elements containing BSA used as a carrier protein.

All isolated specific antibody fractions targeting the three antigens (CMV, BSA, Tg), obtained through both the 2% SLG elution method and a standard acidic buffer approach, were subsequently analyzed on a multi-antigen array (Figure 5). The resulting fluorescent images (Figure 5b–d) were compared against reference images obtained from the analysis of the original sample (Figure 5a). The intensity of the specific signals was compared with that of the original sample (Figure 5g–i). To account for potential nonspecific interactions, control experiments were performed by probing the multi-antigen microarray with the detection antibody alone (goat anti-human IgG; Figure 5f) and with fluorescently labeled anti-BSA antibodies (Figure 5e), with the latter controlling for interference from anti-BSA antibodies in the immunoassay.

**Figure 6:**
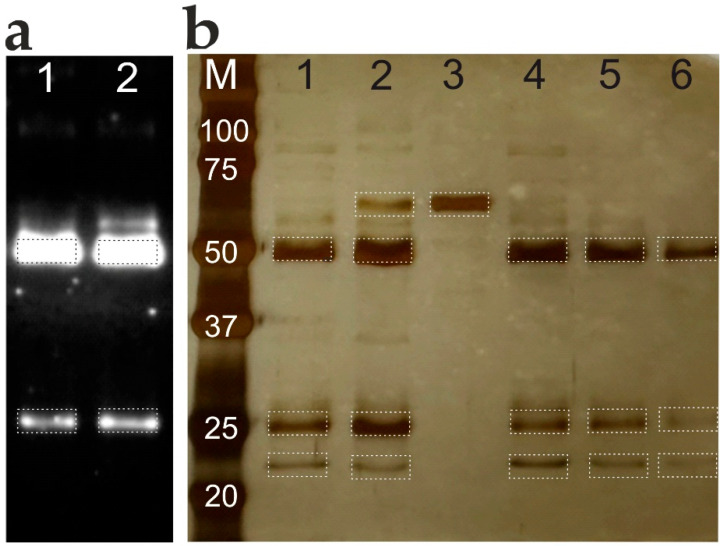
Comparative analysis of total IgG isolated from serum samples using acidic and SLG elution from Protein G magnetic beads. (**a**) Immunoblot analysis under 2% SLG (Lane #1) and acidic elution (Lane #2) conditions. (**b**) Total protein profile visualized by silver nitrate staining. Lanes: (M) molecular weight markers (values in kDa); (1) sample #1, SLG elution; (2) sample #1, Gly-HCl buffer (pH 2.5) elution; (3) human serum albumin reference; (4) sample #2, SLG elution; (5) sample #3, SLG elution; (6) sample #4, SLG elution.

To further validate the broad applicability of the method, the mild anionic detergent elution protocol was adapted to isolate total immunoglobulin G (IgG) from blood sera by using commercial magnetic beads conjugated with Protein G. The elution method’s efficacy was evaluated by comparing the total IgG fractions isolated via the standard acidic buffer with those obtained using the 2% SLG protocol. The integrity and purity of the isolated IgG fractions were subsequently analyzed via electrophoresis and Western blotting (Figure 6).

Analysis of the total IgG fractions isolated from blood sera via electrophoresis revealed distinct bands at 50 and 25 kDa, corresponding to the molecular weights of reduced IgG (Figure 6b). The bands from SLG elution and acid buffer elution (lanes 1 and 2) show no notable differences, indicating comparable efficiency of the two methods. The presence of an additional weak band at 67 kDa in lane 2, co-migrating with human serum albumin (lane 3), suggests harsher elution conditions with the acidic buffer. Both methods (lanes 1 and 2) showed a faint band at 22 kDa, consistent with the molecular weight of protein G. The efficiency of SLG elution was demonstrated using different blood serum samples (lanes 4, 5, 6). Western blot analysis confirmed the successful isolation of IgG using both acid buffer and SLG elution (Figure 6a).

## 4. Discussion

The biological activity, effector functions, and therapeutic efficacy of IgG antibodies are primarily determined by their subclass and a spectrum of post-translational modifications, particularly glycosylation [18]. Therefore, the isolation of specific immunoglobulins is a critical prerequisite for their precise functional characterization. Affinity chromatography—a cornerstone technique for selectively purifying antibodies—leverages the specific and reversible interaction between an immobilized antigen and its target. The process involves two phases: the capture of the target molecule from the solution by the solid-phase ligand, followed by its elution, which involves disrupting the specific complex to release the purified protein. A critical requirement is that the elution process preserves the protein’s native conformation and biological activity. Various strategies can be employed to disrupt the antigen–antibody complex, including changes in pH or ionic strength and the application of chaotropic agents, organic solvents, high-charge eluents, or competitive elution [19]. The mechanism of the eluting agent is a key determinant, as it can induce either reversible, short-lived unfolding or irreversible denaturation of the antibody.

Acidic buffers, such as glycine-HCl (pH 2.5–3.0), represent a gold standard for target antibody elution in the laboratory due to their availability and versatility. The mechanism involves protonating charged amino acid residues, primarily histidines, within the antigen–antibody interface. This protonation disrupts electrostatic interactions by changing the net charge of the molecule and can also compromise hydrogen bonding and alter the isoelectric point. However, this aggressive approach also perturbs the antibody’s tertiary structure, causing partial unfolding and exposure of hydrophobic regions. Such structural alterations frequently lead to a loss of specific antigen-binding activity and can induce undesirable polyreactivity or aggregation [9,10,11]. These detrimental effects are particularly problematic when the isolated antibodies are intended for functional studies or analytical techniques sensitive to structural integrity.

A critical limitation of current methodologies for the microscale isolation of specific antibodies from serum is the universal reliance on acidic buffers for elution, regardless of the sorbent employed. For example, Madara et al. used polyacrylamide gels to isolate antibodies by electrophoretically separating proteins, immobilizing them in situ with glutaraldehyde, and using the homogenized gel as a microsorbent [20]. In another approach, serum autoantibodies were purified using antigen-bound nitrocellulose membranes excised after Western blotting [5]. Brown et al. developed a high-throughput microscale method to purify antigen-specific antibodies for IgG glycan analysis, utilizing streptavidin-functionalized agarose cartridges conjugated with biotinylated antigens [21]. Similarly, Mendis et al. isolated specific autoantibodies from serum using MBP-fusion protein-coated plates [6]. Although these techniques demonstrate versatility in sorbent design, they share a fundamental drawback: the mandatory use of low-pH elution buffers, which can irreversibly compromise antibody structure and specificity.

To overcome the limitations of conventional elution, we developed a gentle method using anionic detergents to dissociate antibodies from micro-immunosorbents, such as microarrays or magnetic particles. The core of this technique lies in the detergent’s transient, non-denaturing interaction with the antibodies, which, after purification via gel filtration, are recovered with high purity and minimal loss of native conformation.

Detergents are amphiphilic molecules consisting of a long-chain hydrophobic aliphatic tail and a hydrophilic polar head group. This structure enables their hydrophobic moieties to penetrate and disrupt phospholipid bilayers by displacing membrane lipids, making them highly effective for cell lysis [22]. Certain detergents have proven remarkably successful in extracting and refolding recombinant proteins from inclusion bodies [23]. Previous studies confirm, for example, that Sarkosyl is a mild, non-denaturing detergent that solubilizes proteins while maintaining their native structure and function [24]. It was also employed to solubilize patient-derived α-synuclein fibrils [25]. A seminal study by Arakawa et al. demonstrated that SLG could recover up to 100% of the native protein, in stark contrast to SDS, which yielded 0% recovery after solubilization [12]. Inspired by the protein-refolding capabilities of these mild anionic detergents, we investigated their potential as gentle eluents for isolating specific antibodies from human serum.

The anionic detergents evaluated in this study exhibit distinct structural characteristics that govern their interactions with protein structures during antibody elution. While SDS acts as a strong denaturant that typically causes irreversible protein unfolding, both Sarcosyl and SLG function as milder alternatives, capable of solubilizing proteins while maintaining their native conformation and biological activity (Table 1). This fundamental difference in protein–detergent interaction mechanisms directly influences their suitability for antibody isolation applications where preserving structural integrity is paramount.

Analysis of antibody recovery as a function of detergent concentration revealed a distinct optimum for each agent, with efficiency declining at both lower and higher concentrations (Figure 3a). At low concentrations, detergent levels remain below the critical micelle concentration (CMC), preventing effective disruption of the antibody–antigen complex and subsequent antibody release. In contrast, the sharp decline in functional yield observed at concentrations substantially above the CMC is likely attributable to protein denaturation, aggregation, and persistent detergent binding that hinders subsequent purification. The final recovery of functional antibodies under optimized conditions highlighted the stark differences in detergent gentleness: SDS yielded only 17%, Sarkosyl provided 45%, and SLG achieved the highest recovery—78% (Figure 3b). Antibody function was entirely dependent on the recovery step, as eluates not subjected to purification exhibited no detectable specific binding. Following the identification of these optimal concentrations (0.025% SDS, 1% Sarkosyl, 2% SLG), elution time and temperature were further refined (Figure 3c).

The method’s robustness to matrix interference was confirmed by spiking experiments in blood serum. Neither the elution efficiency nor the specific activity of the recovered antibodies was compromised by the serum components, confirming the method’s applicability to complex biological samples (Figure 3d).

The isolation of specific antibodies from human serum using microarrays confirmed high functional recovery, with 58.5–85.3% of specific bioactivity retained. Comparative analysis between initial serum samples and isolated antibody fractions was performed using a multi-antigen microarray containing numerous immobilized proteins, including CMV pp150 and Tg (Figure 2). The results demonstrate that the developed method yields sufficient antibody quantities for downstream applications while maintaining structural integrity and antigen-binding capacity (Figure 4). The presented images offer a qualitative visualization of specific binding patterns and represent a preliminary proof of concept. Specific positive signals from microarray elements containing corresponding antigens confirm successful complex formation for both the original serum sample (Figure 4a) and isolated antibody fractions (Figure 4b–d), verifying preserved bioactivity in the purified eluates. Crucially, the lack of cross-reactivity with non-cognate antigens on the microarray indicates that the SLG-based elution process does not induce polyreactivity in the isolated antibodies.

Despite the limited binding capacity of individual microarray gel elements, the platform offers several compelling advantages. Its primary strength lies in exceptional economic efficiency: the system requires only 6 µg of antigen protein to analyze up to 1000 serum samples, enabling antibody isolation even against rare or expensive targets. Furthermore, antigen immobilization within a polyacrylamide hydrogel matrix stabilizes the protein’s tertiary structure, preserving epitopes in their native conformations for at least one year [26]. For applications requiring higher antibody yields, such as subsequent ELISA, the platform can be scaled by fabricating capture microarrays with increased numbers or larger dimensions of gel elements. In summary, this approach represents an inexpensive, rapid, and versatile platform for parallel isolation and analysis of specific antibodies from multiple samples.

The mild-elution-based antibody isolation method was also successfully adapted to a magnetic particle platform to demonstrate its versatility. Figure 5 presents the analysis of the isolated serum antibodies against Tg, CMV, and BSA captured with magnetic beads and eluted with 2% SLG. While the original sample contained antibodies against all three antigens (Figure 5a), each isolated fraction demonstrated high specificity: anti-Tg (Figure 5b), anti-CMV (Figure 5c), and anti-BSA antibodies (Figure 5d). The multiple interactions observed for anti-BSA antibodies (Figure 5d) are attributed to assay interference, as BSA is commonly used as a stabilizer in commercial protein preparations [15]. This was confirmed by direct analysis with fluorescently labeled anti-BSA antibodies (Figure 5e). The specificity of the detecting antibodies used was also validated in a separate control (Figure 5f). Multiplex analysis confirmed the highly selective extraction of target antibodies, with the isolated antibodies retaining functionality and showing no cross-reactivity against unrelated array antigens. These results demonstrate the reversible nature of the SLG–antibody interaction and underscore the method’s high selectivity.

The method’s specificity was demonstrated using a complex serum sample containing multiple distinct antibodies, including anti-Tg (autoimmune), anti-CMV (viral), and anti-BSA (dietary) targets. This successful parallel isolation confirms the platform’s capability for comparative studies of antibody populations across different categories—enabling direct investigation of interaction specificity between autoantibodies and pathogen-specific and food-specific antibodies within the same experimental framework.

The mild anionic detergent elution method was further validated for isolating total IgG from human serum using Protein G-conjugated magnetic beads. Standard acidic elution (Gly-HCl, pH 2.5) and mild elution with 2% SLG were directly compared. Western blot analysis confirmed successful IgG isolation with both methods (Figure 6a), while silver-stained gels demonstrated comparable purity, with minimal co-elution of serum proteins such as albumin (Figure 6b). The consistent band patterns and high purity across multiple serum samples establish SLG elution as a robust alternative to conventional acidic conditions.

Consequently, this strategy enables the selective isolation of target antibodies through mild anionic detergents and versatile immunosorbents, yielding high-purity preparations with preserved structural integrity.

## 5. Conclusions

This study introduces a novel methodology for the microscale isolation of specific antibodies from the complex matrix of human serum. The core innovation of this strategy lies in the synergistic combination of two key elements: the use of antigen-functionalized micro-immunosorbents (hydrogel arrays or magnetic beads) for target capture and a uniquely gentle elution strategy employing anionic detergents to dissociate antigen–antibody complexes without compromising native antibody structure and function. The presented approach offers significant value by overcoming the conventional trade-off between isolation specificity and antibody integrity. The results confirm that this method enables the highly selective isolation of serum antibodies, including autoantibodies, virus-specific antibodies, and antibodies to a food protein, yielding preparations with minimal impurities. Critically, the isolated antibodies exhibit no detectable cross-reactivity in multiplex assays and retain full structural and functional integrity, a direct and innovative outcome of the tailored elution protocol. This robust platform provides a versatile, reliable method for obtaining purified bioactive antibodies, advancing downstream applications.

## Figures and Tables

**Table 1:** Comparison of anionic detergents’ properties.

	SDS (Strong Denaturant)	Sarcosyl (Mild)	SLG (Mild)
Chemical structure	CH_3_-(CH_2_)_11_- -O-SO_3_^−^ Na^+^	CH_3_-(CH_2_)_10_- -C(O)-N(CH_3_)-CH_2_-COO^−^ Na^+^	CH_3_-(CH_2_)_10_- -C(O)-NH-CH(COO^−^)-(CH_2_)_2_-COO^−^ Na^+^
Alkyl tail length	Longest	Shorter	Shorter
Head group polarity	Least polar	More polar	Most polar
Interaction with proteins	Strong, Aggressive	Weaker, Gentle	Weakest, Gentle
Effect on protein structure	Irreversible denaturation	Preserves native structure	Preserves native structure
Known use	Complete unfolding SDS-PAGE, Western blot stripping	Gentle solubilization Inclusion bodies protein extraction	Gentle solubilization Inclusion bodies protein extraction

## Data Availability

The authors confirm that the data supporting the findings of this study are available within the article and/or its Supplementary Materials.

## Supplementary Materials

The following supporting information can be downloaded at https://www.mdpi.com/article/10.3390/bios16010022/s1, Figure S1: Capture array layout. Abbreviations: Ag—immobilized antigen, PBS—Empty gel elements without any protein. For Capture array the following proteins were used for immobilization: bovine serum albumin (A7030, Sigma, St. Louis, MO, USA); recombinant thyroglobulin (8RTG4, HyTest, Turku, Finland); recombinant cytomegalovirus pp150 protein (RE003, Xema Co., Ltd., Moscow, Russia); recombinant human insulin protein (ab123768, Abcam, Cambridge, UK). Table S1: Protein Panel for the Multi-Antigen Array.

## Abbreviations

The following abbreviations are used in this manuscript:

Ab	Antibody
Ag	Antigen
BSA	Bovine serum albumin
CMV	Cytomegalovirus
Ig	Immunoglobulin
INS	Insulin
Sarcosyl	Sodium lauryl sarcosinate
SDS	Sodium dodecyl sulfate
SDS-PAGE	Sodium dodecyl sulfate–polyacrylamide gel electrophoresis
SLG	Sodium lauroyl glutamate
Tg	Thyroglobulin
