# Supplementary material for: Anionic Detergents as Eluents for Microscale Isolation of Antigen-Specific Serum Immunoglobulins
Source: Biosensors (Basel). 2025 Dec 28;16(1):22. doi: 10.3390/bios16010022 (PMC12839167; doi:10.3390/bios16010022)
Supplement: Supplementary file 1 [file biosensors-16-00022-s001.zip › biosensors-4025280-supplementary.pdf]

Supplementary Materials

Anionic Detergents as Eluents for Microscale Isolation of Antigen-Specific Serum Immunoglobulins

Dmitry Trukhin[1], Marina Filippova [1], Alla Tskaeva [2], Ekaterina Troshina [2], Dmitry Gryadunov [1]andElena Savvateeva [1,*]

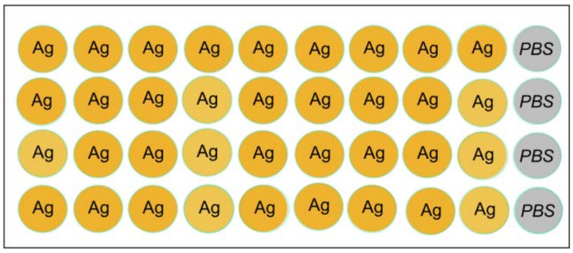

Figure S1. Capture array layout. *Abbreviations:* Ag – immobilized antigen, PBS – Empty gel elements without any protein. For Capture array the following proteins were used for immobilization: bovine serum albumin (A7030, Sigma, St. Louis, MO, USA); recombinant thyroglobulin (8RTG4, HyTest, Turku, Finland); recombinant cytomegalovirus pp150 protein (RE003, Xema Co., Ltd, Moscow, Russia); recombinant human insulin protein (ab123768, Abcam, Cambridge, UK).

Table S1. Protein Panel for the Multi-Antigen Array

Protein	Abbreviation	Catalog #	Source
Marker element	M	-	-
Immunoglobulin A	IgA	31148	Thermo
Immunoglobulin G1	IgG	31R-1087	Biosynth
Immunoglobulin M	igM	16-16-090713	Athens Research
Human serum albumin	HSA	A1653	Sigma
Bovine serum albumin	BSA	A7030	Sigma
Albumin from porcine serum	PoSA	A1830	Sigma
Empty gel	PBS	-	-
Empty gel	PBS	-	-
Interferon alpha 1	IFNa1	228-10814	Raybiotech
Interferon alpha 2a	INFa2a	11100-1	BPL Assay Science
Interferon omega	IFNω	300-02J	PeproTech
Interferon omega	IFNω	BMS304	Thermo
Interleukin 22	IL-22	200-22	PeproTech
Tumor necrosis factor alpha	TNFα	PSG250	Sci-Store
Tumor necrosis factor beta	TNF-β	300-01B	PeproTech
Fibroblast growth factor 2	FGF2	PSG060	Sci-Store
Interleukin 2	IL-2	PSG210	Sci-Store
Interleukin 4	IL-4	PSG040	Sci-Store
Interleukin 6	IL-6	PSG180	Sci-Store
Interleukin 8	IL-8	208-IL	RD Systems
Interleukin 15	IL-15	PSG220	Sci-Store
Interleukin 18	IL-18	B001-5	MBL
Interleukin 21	IL-21	PSG260	Sci-Store
Fc fragment from papain-digested human IgG (heavy chain dimer)	RF	ATF01-01	Arotec Diagnostic
Peptidylarginine Deiminase 4	PAD4	10500	Cayman Chemical
Carbamylated Human Fibrinogen	Ca-Fib	21370	Cayman Chemical
Citrullinated Vimentin	MCV	21942	Cayman Chemical
Double stranded DNA	dsDNA	ATD01-10	Arotec Diagnostic
sIL-6 Receptor α, CD126	CD126	200-06-RC	PeproTech
sIL-6 Receptor α, CD126	CD126	ab167742	Abcam
C-reactive protein	CRP	8CR8	HyTest
Lipopolysaccharide binding protein	LBP	870-LP-025/CF	R&D Systems
Serum amyloid A1	SAA	8SA1	HyTest

Cytochrome P450c21	21-OH	ab225641	Abcam
Cytochrome P450c21	21-OH	CSB-EP006400HU	Cusabio
Cytochrome P450c21	21-OH	ab225641	Abcam
Cholesterol side-chain cleavage enzyme	P450scc	MBS948952	MyBioSource
Ca-sensing receptor	CaSR	CSB-EP004558HU	Cusabio
Glutamic acid decarboxylase 65 kDa	GAD-65	228-20881	Raybiotech
Insulin human	INS	11376497001	Roche
Insulin human	INS	ab123768	Abcam
Insulin receptor	CD220	1544-IR-050/CF	R&D Systems
Tetraspanin-7	TSPAN7	CSB-EP025165HU	Cusabio
Islet cell autoantigen 1	ICA	CSB-EP010947HU(F1)	Cusabio
Tyrosine phosphatase like autoantigen	IA-2	ab42590	Abcam
Thyroid peroxidase	TPO	8RTPO	HyTest
Thyroid peroxidase	TPO	R131	Xema Co., Ltd
Thyroglobulin	Tg	R132	Xema Co., Ltd
Thyroglobulin	Tg	8TG52	HyTest
Thyroglobulin	Tg	8RTG4	HyTest
Thyroglobulin	Tg	8RTG4	HyTest
Tissue transglutaminase 2	TGM2	4376-TG	R&D Systems
Gastric Intrinsic Factor	GIF	abx166676	Abbexa
Alpha subunit of the parietal cell H+/K+-ATPase	ATP4A	abx065478	Abbexa
Beta subunit of the parietal cell H+/K+-ATPase	ATP4B	CSB-EP002343HUe1	Cusabio
Dopachrome delta-isomerase	DCT	abx166564	Abbexa
Keratin 16	KRT16	abx067610	Abbexa
Trichohyalin	TCHH	abx166773	Abbexa
Cytomegalovirus pp150 Protein	pp150	RE003	Xema Co., Ltd
